# The expanded theory of planned behavior for energy saving among academics in Romania, Bulgaria, Turkey, and Slovakia

**DOI:** 10.1038/s41598-025-86795-1

**Published:** 2025-01-22

**Authors:** Silvia Puiu, Sidika Ece Yilmaz, Mihaela Tinca Udriștioiu, Janka Raganova, Zhelyazka Raykova, Hasan Yildizhan, Arman Ameen

**Affiliations:** 1 Department of Management, Marketing and Business Administration, Faculty of Economics and Business Administration, University of Craiova, 200585 Craiova, Romania; 2 Career Planning Application and Research Center, Adana Alparslan Türkeş Science and Technology University, 46278 Adana, Turkey; 3 Department of Physics, Faculty of Sciences, University of Craiova, Craiova, Romania; 4 Department of Physics, Faculty of Natural Sciences, Matej Bel University, 97401 Banská Bystrica, Slovakia; 5 Department of Educational Technologies, Faculty of Physics and Technology, University of Plovdiv Paisii Hilendarski, 4000 Plovdiv, Bulgaria; 6 Energy Systems Engineering, Engineering Faculty, Adana Alparslan Türkeş Science and Technology University, 46278 Adana, Turkey; 7 Department of Building Engineering, Energy Systems and Sustainability Science, University of Gävle, 801 76 Gävle, Sweden

**Keywords:** Energy-saving behaviour, Energy-saving intention, Organisational factors, Organisational identification, Organisational climate, Organisational support, Human behaviour, Environmental sciences, Energy and society

## Abstract

Given the escalating global energy consumption and the concurrent economic and energy crises, energy-saving behaviour must be adopted on a large scale. Universities that are energy-intensive institutions should be one of the institutions where energy-saving behaviour is widely adopted. Academics devote a substantial portion of their time to their offices, which leads to increased energy usage. However, no study has investigated academics’ energy-saving behaviours in the literature. Most studies focus on students or employees in various organizations. Our study tries to cover the gap by examining the energy-saving behaviour of academics in four countries (Romania, Bulgaria, Turkey, and Slovakia) based on the expanded Theory of Planned Behaviour. A questionnaire was distributed to 228 academics from the four countries to gather data. The research hypotheses were tested using partial least squares structural equation modelling. The findings show that individual factors (attitude and perceived behaviour control) influence the energy-saving intention of academics but not the organisational factors due to the weak identification with their universities. The study offers valuable insights for policymakers seeking to promote energy-saving programs in academic institutions. The academics can be seen as role models for their students which emphasizes the need to study more their sustainable behaviours.

## Introduction

The concept of energy grew significantly in Europe through the European Green Deal. Numerous European research initiatives are underway to transition towards a more environmentally sustainable energy system. A key objective of these studies is to enhance energy efficiency in industry and buildings^1^. Furthermore, the repercussions of the energy crisis that plagued Europe in 2021 persist today. The energy crisis, characterised by escalating energy prices and market instability, remains a significant cause for apprehension^2^. These factors contribute to the growing prevalence and implementation of terminologies such as reducing energy consumption, energy efficiency, and energy-saving behaviour (ESB). The impact of energy consumption on economic and social progress and the enhancement of quality of life is of great significance^3^. As a result, the significance of energy savings has progressively heightened. Implementing energy-saving measures is crucial for promoting economic and social progress and maintaining a sustainable environment^4^.

Furthermore, saving energy offers substantial advantages to individuals and organizations^4^. Plus, with the advantages of energy saving, debates exist around its implementation. While some individuals argue that energy saving is an expensive endeavour, others contend that it may be accomplished through simple means. Although energy-saving may involve using expensive components, individuals can also adopt straightforward daily practices, such as switching off lights upon exiting a room, to save energy^5^. For instance, in an organizational setting, individuals can significantly contribute to saving energy by implementing simple actions. The energy-efficient actions of individuals in the workplace reduce air pollution and carbon emissions^6^.

Since organizations and their employees are widely recognised as significant consumers of global energy resources, it is imperative to advocate for the adoption of energy-efficient practices within large organizations^7^. Educational buildings, as one type of large organizations, contribute notably to energy consumption and can exhibit inefficient energy usage patterns^8^. Universities are known as prominent institutions that consume a substantial quantity of energy globally^9^. University World News^10 ^reports a significant surge in energy costs for universities across Europe, emphasising the need for both public authorities and higher education institutions to respond. This news indicates that universities are currently facing an energy crisis. According to the University World News^10^, the National Association of French Universities would face an additional expense of €100 million in 2022. A study on the electricity consumption of the University of Castilla-La Mancha in Spain, specifically focusing on electricity consumption in universities, revealed that the electricity consumed fluctuates monthly. The highest recorded electricity consumption, in July, was 303,795 kWh^11^. These reports and studies indicate that universities are among the significant institutions requiring examination regarding energy consumption. Thus, it is imperative to conduct investigations that delve into the origins of energy consumption.

A crucial aspect that constitutes attention is the proactive involvement of individuals in energy consumption. Energy usage is significantly impacted by individual consumption. Even if this topic is extensively studied in the general population and various organizations, there is a research gap in studying the energy-saving behavior of academics in higher education institutions. Another element of the research gap that was identified is related to studies for academics’ sustainable behavior in universities from several countries. Most previous studies included only students in their research samples^9,12–15^. Several papers incorporate both students and university employees in their samples^16–18^. The results show that academics’ energy-saving behavior has not been studied exclusively. Performing energy-saving research at universities is a cost-effective method that may reduce carbon emissions. The literature evaluation reveals that academics as a sample are rarely studied, and that the studies mostly employ students. Academics frequently spend a substantial amount of time in their offices, deeply engaged in numerous tasks and duties^19^. The fact that they spend a significant amount of time in their offices may be closely related to their energy use and may directly affect this situation. In light of the limited studies focusing exclusively on academics, this research aims to examine their energy consumption patterns. Furthermore, no research has analysed academics’ environmental attitudes, intentions, and behaviors in European countries. The present study seeks to address this gap in the existing body of research. The theoretical implications of our research refer to the opportunity for other researchers to look more into the academics’ behavior for energy saving in universities from different countries, considering the position of these professionals as role models for the younger generations they are interacting with. The practical implications consider the usefulness of our findings because they can shape the strategic decisions of policymakers in universities, generating multiple changes in the way academics perceive the importance of adopting sustainable behaviors in universities.

Moreover, the studies defined psychological, social, socio-demographic, and situational factors as predictors of ESB^20,21^. However, it should be noted that organizational factors might also impact individuals’ willingness to save energy in addition to these other aspects. For instance, an individual’s pro-environmental actions are correlated positively with their sense of belonging to the company^22^. Also, organizational support (OS) significantly affected whether people were willing to save energy at work^23^. Furthermore, one of the key factors influencing environmental behavior is a sense of belonging^24^. Such aspects must be looked at in the context of organizational considerations. Additionally, the promotion of energy-saving studies in public institutions is demonstrated to depend heavily on organizational behavior. It contributes to developing the organizational climate (OC) regarding interdepartmental relationships and the institution’s favourable perception of energy savings^25^. The challenge of energy saving is influenced by organizational factors such as bottom-up energy-saving strategies, the culture of energy conservation, and comparative energy feedback among departments^26^.

The Theory of Planned Behavior (TPB) model is widely used for pro-environmental behavior^18,27–29^. The components that define intention and turn it into behavior, corresponding to the TPB model, include attitude toward behavior (ATT), subjective norms (SN), and perceived behavioral control (PBC)^30^. The TPB approach, as previously employed, lacks consideration for organizational factors. It is considered reasonable to adopt the TPB as the fundamental theoretical framework in this study for comprehending the ESB of academics by extending the TPB model with organizational factors.

This study contributes to the existing literature on ESB in different ways. Previous research on ESB has predominantly centred around students, with limited emphasis on academics. The study used academics as the sample population. Additionally, it is worth noting that previous research on ESB has primarily concentrated on examining the factors that influence intention, utilising the TPB model. The present study incorporated environmental concern (EC) and environmental knowledge (EK) as additional variables into the model and subsequently examined their impact on attitude. The study employed the extended TPB model to investigate organizational factors for the first time. The study demonstrated the academics’ environmental attitudes, intentions, and behaviors in four European countries.

Starting from the literature gap on studies addressing exclusively the academics’ energy-saving behavior in universities, this research focused on several objectives of identifying: the knowledge of academics towards environmental matters and their interest and concern in adopting more sustainable behaviors; their intention to adopt such behaviors; the influence of subjective and organizational factors in their decisions; their general attitudes towards these issues and the control they feel they have in making their decisions. The originality of this research consists of applying an extended model of the Theory of Planned Behavior for a category that has not been extensively studied. To achieve this, partial least squares structural equation modeling was employed, incorporating the variables of the extended TPB model as constructs.

The following outlines the paper’s structure: Section 2 studies pertinent content and creates the conceptual framework. Section 3 describes the survey’s design, the procedure for gathering the data, and the method used for data analysis. In Section 4 the results are presented, and Section 5 addresses and highlights findings and consequences. The final section concludes and outlines possible future directions.

## Literature review

The Theory of Planned Behavior is commonly employed in research to examine the relationship between individuals’ behavioural intentions and their subsequent actual behavior^18,31–33^. Attitude toward behavior, subjective norms, and perceived behavioral control influence behavioral intention, determining the actual behavior^30^. As pro-environmental workplace behaviors have grown in popularity, studies have employed the original or expanded TPB models to predict energy-saving actions^32^. This study extends the TPB model by including organizational factors in addition to the three fundamental factors used to predict behavioral intention.

### Environmental concern

The concept of environmental concern pertains to an individual’s level of awareness and consciousness of environmental issues, as well as their degree of support for efforts to address these issues^34^. It describes the extent of individuals’ tendency to acknowledge and endorse the solutions to ecological issues^35^. The level of environmental concern is predictive of individuals’ willingness to engage in pro-environmental practices^36^. Dunlap and Jones^34^ mention the need to integrate environmental issues in organizations, considering the role environmental interest plays from a sociological point of view. Ru et al.^6^ studied the impact of environmental concern on the attitudes toward energy-saving behavior among employees in several Chinese companies, with their findings revealing a positive correlation between the variables. The following hypothesis was developed starting from the literature review and the Theory of Planned Behavior: H_1_: Environmental concern has a significant influence on the attitudes toward energy-saving behavior.

### Environmental knowledge

Environmental knowledge has been viewed as a fundamental understanding of the facts, concepts, and relationships pertaining to the natural settings and the surroundings^37^. It strongly influences pro-environmental behavior^38^. Individuals with a high level of EK have been considered to have a favourable and high attitude toward behavior for saving energy. Previous research has demonstrated a statistically significant and consistent correlation between EK and ATT^39–41^. Fryxell and Lo^37^ show that environmental knowledge has an important impact on the attitudes of managers to initiate a specific behavior. Li et al.^41^ studied the impact of environmental knowledge on the attitudes of individuals in a household to buy more eco-friendly appliances for their homes. The literature shows significant relationships between EK and ATT which impacts individual decisions to adopt a specific behavior. Based on these findings and the TPB model, a second hypothesis was developed: H_2_: Environmental knowledge has a significant influence on the attitudes toward energy-saving behavior.

### Attitudes toward energy-saving behavior

Attitudes play a significant role in determining an individual’s behavioral intentions. If an individual has a favourable attitude toward energy-saving behavior, the likelihood of engaging in that behavior will be high^30^. It has been suggested that individuals with a favourable attitude towards saving energy, recognising its significance in mitigating air pollution and promoting a sustainable environment are more likely to exhibit a stronger inclination toward engaging in energy-saving behavior^6^. Employees’ ESB depends on their attitudes^42^. Xie et al.^42^explain the positive correlation between these two variables from a psychological perspective, emphasizing the importance of understanding it in order to implement effective strategies in an organizational context. Research findings have demonstrated a statistically significant correlation between attitudes and intention^33,42,43^. Fatoki^43^ validated the positive relationship between these variables in a study focused on the willingness of employees to save energy at the office. Therefore, it is hypothesised that individuals with a favourable attitude toward energy savings will have higher energy-saving intentions. Considering the relevant literature and the TPB model, a third hypothesis was developed: H_3_: Attitudes toward energy-saving behavior have a significant influence on energy-saving intentions.

### Perceived behavior control

Perceived behavior control refers to an individual’s subjective assessment of the level of ease or difficulty associated with engaging in a certain behavior^30^. It plays a significant role in shaping an individual’s intention to engage in energy-saving intentions (ESI). ESI is influenced by a favourable sense of behavioral control. This is because people frequently have no control over things like cost, time, skills, etc^44^. Individuals who possess self-control, experience a sense of autonomy within their work settings, and possess the requisite information, skills, and time related to saving energy are more likely to exhibit a desire to save energy. Previous studies have indicated a statistically significant correlation between PBC and intention^33,42,45^. Ajzen^30^ researched the TPB and identified that PBC is a predictor for the intention of engaging in a specific behavior. Ru et al.^44^ found that PBC was the most important predictor for energy-saving intentions. The literature review on the TPB model and its application to environmental issues formed the development of the fourth research hypothesis: H_4_: Perceived behavior control has a significant influence on energy-saving intention.

### Subjective norms

According to Ajzen^30^, subjective norms refer to an individual’s perception of the beliefs held by others about their actions. The behavioral intentions of individuals can be influenced by the opinions and judgments of significant individuals within their social circle^46^. If an individual perceives that their colleagues and management within their immediate surroundings expect them to save energy, they may demonstrate a willingness to act accordingly. Several studies have found a statistically significant correlation between SN and intention^28,33,42,45,47,48^. Ly and Ly^28^identified that subjective norms are an important factor that impacts the intention of employees to save energy at work. Chen^45^ found that both attitudes and subjective norms are significant predictors of the intention of people to reduce their carbon footprint and thus mitigate climate change. The application of the TPB model in the literature guided the formulation of the fifth hypothesis: H_5_: Subjective norms have a significant influence on energy-saving intentions.

### Organizational factors

Social Exchange Theory is a significant theoretical framework employed in the examination of employee behaviors^49^. The relationship between employees and organizations can be conceptualised as a social exchange relationship. Organizations facilitate the process of social change by acknowledging and appreciating the contributions made by their employees to the overall functioning of the business. Consequently, employees reciprocate by exhibiting behavior that aligns with the organization’s expectations and objectives^50^. Presumably, energy-saving behavior can be considered a social exchange. If organizational identification (OI) and organiztional support (OS) exhibit high levels, and if organizational climate (OC) is positive for pro-environmental behaviors, then it is more probable that employees will engage in energy-saving behaviors.

Once an appropriate indication of intention has been discovered, it will yield the most accurate behavior prediction^30^. Hence, it is imperative to investigate the various aspects that could potentially influence intention on a broad scale. Organizational factors may also influence the ESI and ESB of individuals in the workplace. In this study, OI, OC, and OS variables are examined as organizational factors.

Organization identification refers to a psychological attachment whereby individuals absorb the distinguishing attributes of the organization as their defining attributes^51^. A favourable correlation exists between employees’ level of identification with the organization and their engagement in environmentally friendly behaviors^22^. Shah et al.^22^found a positive correlation between the employees’ identification with their organizations engaging in corporate social responsibility projects and their intention to be more eco-friendly. A study revealed that OI has a positive impact on ESI^18^. Heib et al.^18^ studied the correlation for a sample consisting of both students and employees of universities in Germany and found a positive relationship, but they emphasised that it was weaker than that between subjective norms and intention. Another study by Leygue et al.^52^ demonstrated a significant relationship between OI and ESI. It is proposed that the greater an individual’s identification with the organisation, the greater their ESI. These studies led to the development of the sixth hypothesis related to the extended TPB model: H_6_: Organizational intention has a significant influence on energy-saving intention.

Organizational climate addresses individuals’ perception of the psychologically significant elements within the work environment. It has been proven that OC can impact employees’ behavior in the workplace^53^. OC is acknowledged as a crucial situational factor that impacts employee attitudes^54^. The findings of a study show that the establishment of an electricity-saving environment within an organization is likely to result in employees developing a favourable attitude toward saving electricity and actively engaging in such endeavours^55^. Studies showed that organizational electricity-saving climate positively affected electricity-saving intention^56,57^. Fatoki^56^ applied an extended TPB model by adding organization climate among the factors that influence significantly the energy-saving intention of employees. Wu et al.^57^ found a positive correlation between organizational climate and the employees’ intention to save electricity at work. Another study conducted by Lo et al.^58^ examined energy-saving behavior within the context of an organization across four distinct organizations. The findings revealed notable variations in the four behaviors among these organizations. This disparity may arise from variations in the OC among various organizations. Therefore, it is imperative to investigate the impact of the OC variable on intentions and behaviors related to saving energy. Therefore, if the OC in the workplace encourages pro-environmental behaviors and develops policies to support them, it is anticipated that the employees will be influenced by this climate. It is expected that individuals in organizations with a pro-environmental climate will have ESI. These studies contributed to the development of the seventh hypothesis: H_7_: Organizational climate has a significant influence on energy-saving intention.

Organizational support corresponds to the employees’ perception of the extent to which the organization places significance on their efforts and demonstrates concern for their overall well-being^50^. According to a study conducted by Xu et al.^23^, there was a notable primary impact of OS on the willingness to save energy in the workplace. The provision of management support is a crucial element within the framework of OS^23^. The studies showed that the top management has a favourable correlation with the ESB^59^and ESI^60^. Nguyen and Tran^60^studied the role played by managers in determining a higher intention from their employees to behave in a more sustainable way at work and thus protect the environment. The results showed a positive correlation which is useful for policymakers in organizations. Accordingly, it is believed that high organizational support will result in a high energy-saving intention. Considering the studies on the extended TPB^23,59,60^, the eighth hypothesis was established: H_8_: Organizational support has a significant influence on energy-saving intention.

### Energy saving intention

The likelihood of individuals engaging in energy-saving behavior is positively correlated with their intentions to execute such behaviors^61^. It has been demonstrated that behavioral intention is a significant predictor of actual behavior^62^. The actual ESB of individuals within the workplace was significantly influenced by behavioral intention^63^. Arya and Chaturvedi^33^validated the hypothesis according to which the intention to save energy leads to the actual behavior which emphasized the need to start from developing employees’ intentions in the organizations where they work. The existing literature demonstrates a statistically substantial and consistent correlation between ESI and ESB, as evidenced by many studies^18,33,43,48^. Starting from previous studies on the TPB model and their findings, the ninth hypothesis was developed: H_9_: Energy-saving intention has a significant influence on energy-saving behavior.

A statistically significant positive relationship exists between organizational support and energy-saving intentions^23^; organizational support and energy-saving behaviors^64^; organizational identification and energy-saving intentions^18 ^and organisational identification and environmentally friendly behaviors^22^. There is a marginally significant relationship between organisational electricity-saving climate and electricity-saving behavior^65 ^and a significant correlation between organisational electricity-saving climate and electricity-saving intention^56^. Accordingly, the existing literature suggests that there are correlations between the variables, addressing the fact that ESI plays a significant role in mediating these correlations. Wang and Zhang^48^ emphasized the positive impact of energy-saving intention on the students’ energy-saving behavior, noting that this relationship is strengthened by organisational factors in their universities. With the addition of the specified variables, it is anticipated that the coefficient of the effect of energy-saving intentions on energy-saving behaviors might decrease or disappear. On this basis and considering the extended TPB model which includes organisational factors, the following three hypotheses were developed: H_10_: Energy saving intention mediates the effect of organisational identification on energy saving behavior; H_11_: Energy saving intention mediates the effect of organizational climate on energy saving behavior; and H_12_: Energy saving intention mediates the effect of organizational support on energy saving behavior.

After carefully reviewing the literature on the topic of energy-saving behavior of academics by searching Google Scholar, Scopus and Web of Science, the authors identified that there is a lack of studies that address this target group. Most studies were on employees in general or on students, and some of them addressed both students and employees in universities. Another gap identified was the lack of studies focused on more countries, most of them being focused on only one country. Addressing these research gaps, this study investigates the energy-saving behavior of academics in four countries through the application of an extended TPB model. Academics are recognized not only as employees within educational institutions but also as influential role models for their students.

## Methodology

**Fig. 1:**
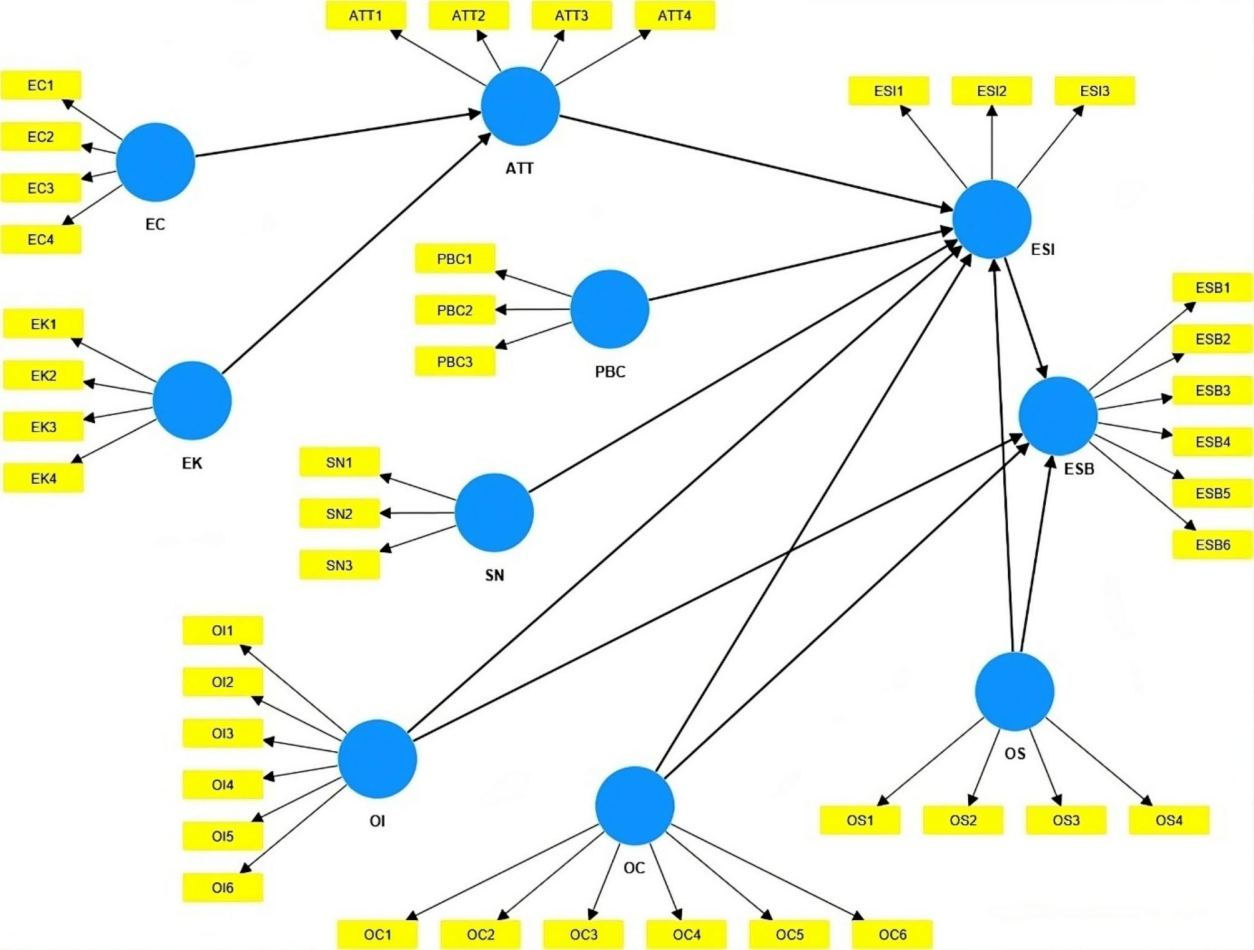
Research Model.

The scales were drawn and revised from previous studies. A fully anchored, five-point Likert-type rating scale (1 = Strongly disagree to 5 = Strongly agree) was used in the study for all items. EK was measured using four items adapted from Wang et al.^66^and Hamzah and Tanwir^67^. OS was assessed with the help of four items modified from Xu et al.^23^ and Xie et al.^42^. The items related to each construct are listed in Table 1 and the proposed model is shown in Fig. 1.

**Table 1:** The constructs and items of the model.

Constructs	Items
OI Mael and Ashforth^68^	When someone criticises my organization, it feels like a personal insult.
	I am very interested in what others think about my organization.
	When I talk about this organization, I usually say ‘we’ rather than ‘they.’
	The organization’s successes are my successes.
	When someone praises this organization, it feels like a personal compliment.
	If a story in the media criticised the organization, I would feel embarrassed.
PBC	I think that I am capable of saving energy in my daily life.
	I have the knowledge and skills to save energy in my daily life.
Ru et al.^44^	Whether or not to save energy is completely up to me.
ESI	I am willing to save energy in my daily life.
Ru et al.^44^	I intend to engage in energy-saving activities in my daily life. I will make an effort to save energy in my daily life.
SN	My colleagues think that I should save energy in my organization.
	My managers think that I should save energy in my organization.
Gao et al.^31^	The high-level management team would want me to save energy in my company.
OC	Others who are important to me have participated in energy-saving behavior.
	My organization encourages energy saving.
	My organization puts much value on energy saving.
	My organization is concerned with becoming more environmentally friendly.
	I regularly see messages about energy saving on social media.
Tang et al.^69^	I regularly see energy-saving messages in newspapers, television, and other media.
ATT	I think saving energy in my daily life is helpful in protecting the environment.
	I think saving energy in my daily life is important to reduce carbon emissions.
	I think saving energy in my daily life is valuable in alleviating energy shortage issues.
Wang and Zhang^48^	I think saving energy in my daily life is a wise action.
EC	I think human beings should protect the environment.
	I am concerned about air pollution.
	I am concerned about the haze problem.
Ru et al.^6^	I am worried about climate change.
EK	I am very knowledgeable about environmental issues.
	Compared to the average person, I am more familiar with issues related to the environment.
Hamzah and Tanwir^67^; Wang et al.^66^	I know energy-saving methods well. I know much about the energy-saving tips in daily life.
OS	I have heard about saving energy or improving energy efficiency from my manager/supervisor, or my colleagues.
	Our organization incentivises energy-saving or energy-efficiency behaviors.
	The organization often organises activities that encourage employees to take energy-saving behaviors, including lectures, training, and knowledge contests.
Xu et al.^23^; Xie et al.^42^	The organization integrates energy-saving elements into the layout of the office environment by posting slogans, tips, and other methods.
ESB	When I am at work in my office, I turn off the lights when going out, even for a short time.
	I reduce the use of the fan/air conditioning by opening the windows.
	I switch off the computer when it is not used.
	I turn off the lights when the sunshine is bright enough.
	I prefer to walk short distances.
Zhang et al.^65^; Arya and Chaturvedi^33^	I prefer to travel by public transport.

The researchers gathered primary data utilising the convenience sampling technique. Quantitative data was collected using a questionnaire method from academics in Turkey, Romania, Bulgaria, and Slovakia. Participants were chosen based on their availability and ease of access. The project members from participating countries collected the data from their countries via online form. Thus, the participants of the study consisted of 228 academics.

## Results

The survey was disseminated online to academics in four countries: Romania, Slovakia, Turkey, and Bulgaria. Compared to other university employees, academics were chosen to be the target group of this study because they shape future generations’ minds, attitudes, beliefs and habits, representing models for students. Four universities from four different countries worked together in an Erasmus + project. The project was the framework to apply the survey and to work together. The project team received 228 valid questionnaires with the following distribution: 35.5% from Romania, 23.2% from Bulgaria, 22.4% from Turkey and 18.9% from Slovakia. 57% of the respondents were women; regarding their distribution by age, most of them were between 40 and 49 years old (32%), followed by those between 31 and 39 years old (27.2%), between 50 and 59 (24.6%).

**Fig. 2:**
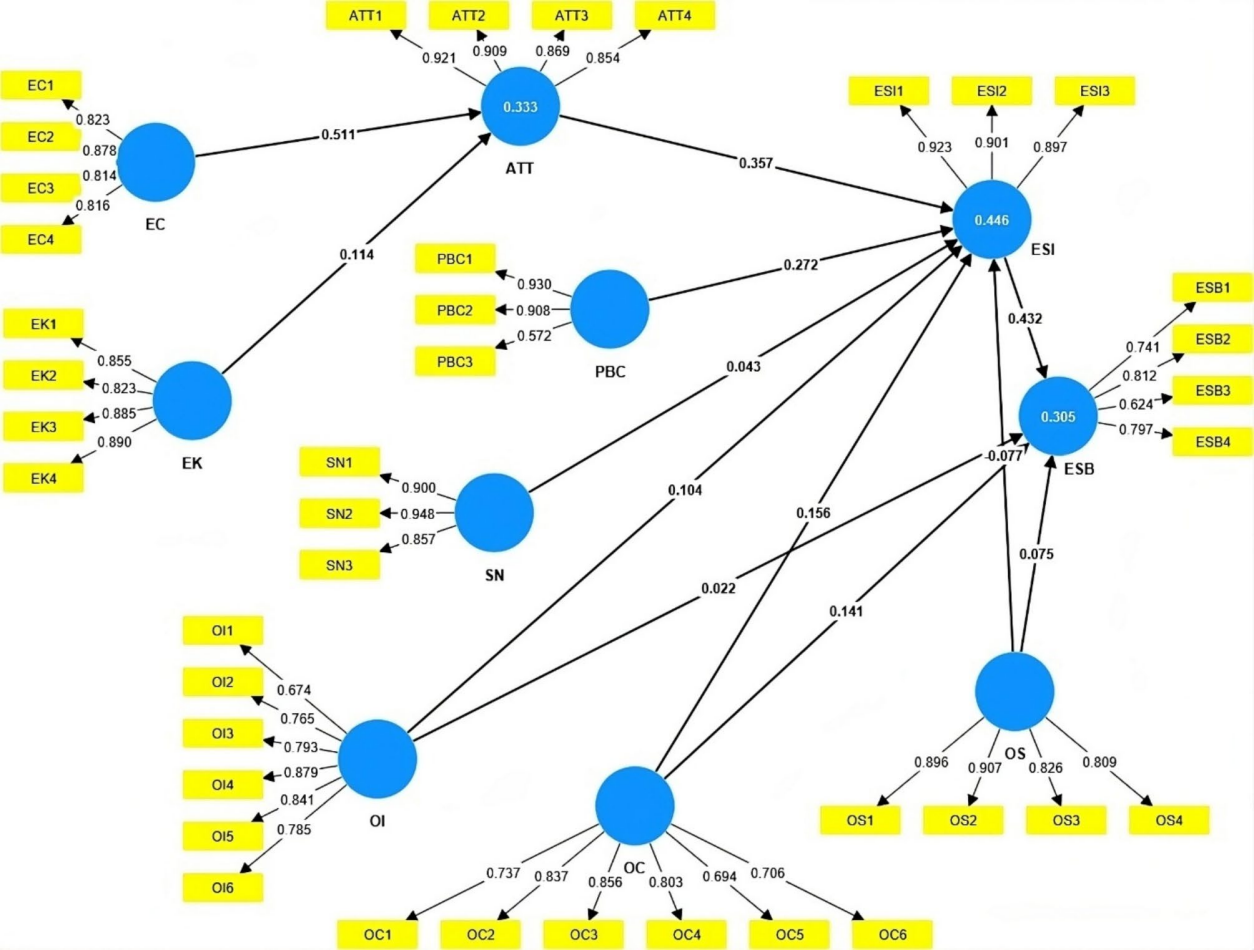
The research model after removing items with low outer loadings.

Table 2shows the outer loadings and variance inflation factor (VIF) for the items in the model. Outer loadings between 0.5 and 0.7 are acceptable, and those above 0.7 show high reliability; meanwhile, VIF values below 5 do not pose collinearity issues between the model variables^70^. It was noticed that ESB5 (I prefer to walk short distances) and ESB6 (I prefer to travel by public transport) have their outer loadings below 0.5. These items demonstrate a poor correlation with the factor and are insignificant in explaining it, so the authors decided to remove them from the proposed model. This could be attributed to different cultural contexts within the sample. Thus, the model changes, as shown in Fig. 2.

**Table 2:** Reliability and collinearity of the model.

Items	Outer loadings	VIF	Mean
ATT1	0.921	4.053	4.478
ATT2	0.909	4.183	4.311
ATT3	0.869	2.665	4.250
ATT4	0.854	2.215	4.452
EC1	0.823	1.834	4.693
EC2	0.878	2.604	4.412
EC3	0.814	2.056	4.083
EC4	0.816	1.783	4.246
EK1	0.855	2.300	3.781
EK2	0.823	2.092	3.860
EK3	0.885	3.538	3.759
EK4	0.890	3.606	3.785
ESB1	0.705	1.344	4.048
ESB2	0.790	1.786	4.189
ESB3	0.617	1.278	3.882
ESB4	0.777	1.631	4.627
ESB5	0.464	1.198	4.37
ESB6	0.349	1.229	3.21
ESI1	0.922	2.927	4.404
ESI2	0.901	2.748	4.167
ESI3	0.897	2.406	4.263
OC1	0.742	1.415	3.750
OC2	0.833	3.052	3.605
OC3	0.853	3.894	3.478
OC4	0.800	2.695	3.579
OC5	0.696	3.797	3.439
OC6	0.708	3.826	3.500
OI1	0.669	1.741	3.276
OI2	0.765	1.973	3.504
OI3	0.797	1.867	3.776
OI4	0.880	2.617	3.640
OI5	0.839	2.545	3.509
OI6	0.784	1.977	3.469
OS1	0.904	2.157	3.224
OS2	0.910	2.710	3.281
OS3	0.812	4.447	2.895
OS4	0.794	4.326	2.982
PBC1	0.930	2.308	4.268
PBC2	0.908	2.246	4.070
PBC3	0.572	1.226	3.711
SN1	0.900	2.534	3.263
SN2	0.948	4.059	3.412
SN3	0.857	2.559	3.548

Table 3 shows that Cronbach’s alpha and Composite Reliability (CR) are higher than 0.7, and the Average Variance Extracted (AVE) is above 0.5 for all constructs in the model, which indicates the high reliability and validity of the proposed model. For the discriminant validity, the Fornell-Larcker criterion is shown in Table 4. It can be seen that the values in the main diagonal of Table 4 are higher than the ones in the same column, showing that the variables are different enough between them.

**Table 3:** Reliability and validity of the constructs in the model.

Constructs	Cronbach’s alpha	Composite reliability (rho_a)	Composite reliability (rho_c)	Average variance extracted (AVE)
ATT	0.911	0.917	0.938	0.790
EC	0.854	0.858	0.901	0.695
EK	0.886	0.888	0.921	0.746
ESB	0.734	0.753	0.833	0.558
ESI	0.893	0.898	0.933	0.823
OC	0.868	0.889	0.899	0.600
OI	0.884	0.923	0.909	0.627
OS	0.896	1.007	0.919	0.741
PBC	0.759	0.889	0.856	0.673
SN	0.887	0.910	0.929	0.815

**Table 4:** Fornell-Larcker criterion.

	ATT	EC	EK	ESB	ESI	OC	OI	OS	PBC	SN
ATT	0.889									
EC	0.569	0.833								
EK	0.371	0.502	0.864							
ESB	0.435	0.393	0.330	0.747						
ESI	0.568	0.502	0.436	0.518	0.907					
OC	0.379	0.314	0.446	0.387	0.429	0.775				
OI	0.299	0.320	0.240	0.253	0.339	0.405	0.792			
OS	0.243	0.222	0.272	0.289	0.249	0.700	0.376	0.861		
PBC	0.469	0.473	0.658	0.430	0.539	0.468	0.297	0.261	0.820	
SN	0.300	0.240	0.313	0.279	0.329	0.538	0.309	0.479	0.368	0.903

The authors applied the bootstrapping test to test the hypotheses, and the results are depicted in Table 5.

**Table 5:** Bootstrapping test for testing hypotheses.

	T statistics	*P* value	Confidence intervals bias corrected	Hypotheses validation
EC -> ATT	6.988	0.000	(0.365, 0.649)	H1 validated
EK -> ATT	1.378	0.168	(−0.055, 0.266)	H2 not validated
ATT -> ESI	4.064	0.000	(0.189, 0.527)	H3 validated
PBC -> ESI	3.587	0.000	(0.126, 0.421)	H4 validated
SN -> ESI	0.748	0.454	(−0.063, 0.165)	H5 not validated
OI -> ESI	1.671	0.095	(−0.028, 0.220)	H6 not validated
OC -> ESI	1.873	0.061	(−0.001, 0.327)	H7 not validated
OS -> ESI	0.975	0.330	(−0.235, 0.083)	H8 not validated
ESI -> ESB	6.125	0.000	(0.287, 0.559)	H9 validated
OI -> ESI -> ESB	1.580	0.114	(−0.008, 0.105)	H10 not validated
OC -> ESI -> ESB	1.924	0.054	(0.003, 0.141)	H11 not validated
OS -> ESI -> ESB	0.971	0.332	(−0.103, 0.035)	H12 not validated

Only four hypotheses were validated: H_1_, H_3_, H_4_, and H_9_, because only for them, t values above 1.96 and p values below 0.05, and the confidence intervals bias corrected do not include zero. The other hypotheses measuring a direct effect (H_2_, H_5_-H_8_) or an indirect effect of mediation (H_10_-H_12_) which were not validated.

## Discussion

The objective of this study was to examine the factors that exert influence on the ESB of academics. To do this, a theoretical framework, the TPB model, is constructed and verified, incorporating organizational factors. The findings are presented and discussed in the context of other studies that researched similar variables of the model.

H_1_was validated, showing that the impact of EC on ATT is strong (0.511), and the academics preoccupied with environmental issues also have specific attitudes oriented towards protecting the environment and reducing their carbon footprint. Other studies showed a positive correlation between environmental concern and attitudes^6,41,46^. Chen and Tung^46^ researched the impact of EC on attitudes toward eco-friendly hotels and found a direct and positive relationship. Thus, they acknowledge that environmentally conscious individuals exhibit a favourable disposition toward saving energy. H_2_was not validated, and the results show that the impact is relatively weak (0.114). In our case, knowing environmental aspects does not inherently mean someone will change their attitudes. Some authors identified a positive correlation^40^, meanwhile others emphasized the impact of culture on the relationship between EK and ATT^71^. Barber et al.^38^ also made the distinction between actual knowledge and the respondents’ self-assessment regarding knowing. The authors point out that real knowledge leads to significant changes in their attitudes. Geiger et al.^71^conducted research on students from Argentina and Germany and found that culture, the economic and the social environment in which the students live influence the impact of knowledge on the environmental attitudes they manifest. The differences noticed in our study might be explained by the fact that we collected data from four countries. Furthermore, when formulating hypotheses, the impact of EK on ATT was considered. Nevertheless, the underlying factors contributing to the outcomes may vary based on the specific circumstances and the behavior of individuals inside the organization. Thus, whereas certain research showed the presence of robust connections^38,39^, this particular investigation revealed that organizational behavior was devoid of significance. H_3_was validated, indicating that favourable attitudes towards environmental protection influence academics’ intention toward energy saving. This finding is in line with the results of other authors^6,28,32,42,43,48^. Wang and Zhang^48^ conducted research on Chinese students and found a positive relationship between their attitudes and their intention to save energy. H_4_was validated, showing that the academics who perceive themselves as having a higher level of control over their behavior are also more inclined to save energy and be more proactive, which corresponds to the findings in other studies^6,18,28,42,44,45,48^. Both Heib et al.^18^and Wang and Zhang^48^ conducted their studies on students and found that having a high PBC leads to an increase in their intention to adopt greener behaviors. H_5_was not validated, and the impact from SN to ESI was weak (0.043), showing that the subjective norms do not exert a sufficient influence on the academics’ intention regarding energy saving. The respondents in the four countries differ in terms of their cultures, partly explaining the differences in perception related to the subjective norms identified among their colleagues or superiors. Still, other studies found a significant influence from SN to ESI^28,33,42,45,47,48^, meanwhile other authors focus on the influence of cultural values on the relationship^72,73^. Alam et al.^72^ appreciate that some cultures are more individualistic than others which can influence the impact of the subjective norms on the intentions to save energy. Jiang et al.^73^ studied Chinese households and concluded that one of the explanations for SN not having a significant impact on ESI is related to cultural differences. Gao et al.^31^studied employees’ behavior at work and found that the impact of SN on ESI was insignificant. Another factor that can contribute to the results’ variation could be the structure of the sample. Thus, a study that selected households in their sample^47^ showed significant relationships while our research found the correlations to be insignificant. The cause of this situation is that, in the survey conducted among households, individuals who possess environmental consciousness, environmental knowledge, and concern and who have peers in their social network who prioritise environmental issues tend to respond to questions following subjective norms. Within an organization, the employee’s sphere of influence is more restricted. The outcomes may be rendered insignificant if the organization has insufficient policies and practices to safeguard the environment or energy. H_6_was not validated, and the impact from OI to ESI was weak (0.104), showing that the identification of the academics with their university does not significantly influence their intention to save energy. Some authors identified a positive correlation between the two variables^18,22,52^, while Kim et al.^74^ highlight that “it is reasonable to question the extent to which these findings would be replicated in other colleges with different histories and cultures.” H_7_was not validated, and the impact from OC to ESI was weak (0.156), showing that the climate in the universities was not powerful enough to generate changes in the academics’ intention to save energy. Other studies found a positive correlation^53,55–58^. Zhang et al.^55^ researched employees in Beijing and validated the impact of organizational climate on the employees’ attitudes and through this mediation on the energy-saving intention of the respondents. If we look at the hypotheses validated in our model, we notice that ESI is influenced by ATT and PBC (both more linked to the individual) but not by SN, OI, or OC, which are more linked to other people or even the entire organization for which they work. The authors Wu et al.^57^ highlight that “when the organizational electricity-saving climate of saving electricity is strong, the effect of a personal norm on intention is weakened.” In our research, the personal factors showed a more powerful impact on ESI than those related to the organization.

As in the case of the other two organizational factors, H_8_ was not validated. Our findings show that the intention of the academics to save energy is not linked to the support received from the universities or the managers in the institution. Even if weak (−0.077), the correlation is also negative, reflecting that the academics do not receive adequate support and motivation from the university to engage in energy-saving behaviors. Still, the academics show individual willingness, as revealed by the validated hypotheses so far. As shown in Table 2, the items of OS have the lowest mean. The respondents do not feel the support of the universities in this direction. Other studies found a positive correlation but they were not conducted for universities or in more than one country^23,59,60^. H_9_was validated, showing that the impact of ESI on ESB is strong (0.432), which was also found by other studies^18,33,43,48,61,63,75^. This means that the intention of the academics in the universities of the four countries to save energy leads to pro-environmental behavior on their part, the personnel being proactive. The other studies conducted in universities^18,48^ showed similar findings with ESI being the most important factor that leads to ESB. Neither of the hypotheses H_10_, H_11_, and H_12_ were validated in our study. Thus, the direct effect of the organizational factors OI, OC, and OS on ESI was not noticed (H_6_, H_7_ and H_8_ were not validated), so it was unsurprising to find that the mediation of the academics’ intention on their behaviors does not change the situation. As we see in Table 2, the items for organizational factors have the lowest mean, which shows a lack of identification with their university, a lack of support, and a weak climate, which do not encourage the personnel to engage in green behaviors, at least not directly. Other studies found direct correlations between OS and ESI^23^, OS and ESB^64^, OI and ESI^18^, OC and ESB^65^and OC and ESI^56^. We did not find studies where organizational factors influence ESB, being mediated by ESI.

The added value of our research consists of applying the extended TPB model to the academics in four countries which share some cultural similarities which might explain why other studies reached different findings for the correlations between some of the variables^71^. This study also showed that for the academics in these universities, personal factors are more powerful in influencing their energy-saving behavior than the organizational factors due to weak organizational cultures.

## Conclusion

The study aimed to investigate the factors that influence the ESB of academics. TPB provides a solid framework for elucidating ESB, and the study attempted to enhance the TPB model by incorporating organizational factors. This study contributes to the corpus of knowledge on ESB by providing in-depth insight into organizational factors that were frequently overlooked in prior research and had not been considered in the analysis. Policymakers and practitioners exhibit an acute concern in comprehending energy use within the workplace and devising strategies to mitigate it^76^. In summary, the following key findings were identified in this study:

- Environmental concern, but not environmental knowledge, led to pro-environmental attitudes among the academics.
- Energy saving behavior of academics is strongly influenced by the attitudes and the personal behavior control that the academics feel having.
- Energy-saving behavior is strongly influenced by the academics’ intention to adopt greener behavior at work.
- Neither social norms nor organizational factors showed significant influence on the energy-saving intention or the energy-saving behavior.

Several potential explanations exist for the lack of significant effects of organizational factors in the applied model. For instance, the obtained outcomes may lack significance owing to variations in data originating from diverse countries, disparities in cultural and geographical factors, and disparities in economic circumstances. These variations have the potential to alter the influence of organizational factors. Furthermore, it is essential to note that the impact of organizational factors might vary as time progresses. Given that the research was conducted during a certain timeframe, it is plausible that the influence of these factors may have been relatively limited within that particular era. The influence of organizational factors might be contingent upon the culture and structure of the organization. Hence, incorporating organizational culture within the research process can yield significant findings in studies conducted inside institutions with comparable cultural contexts. Moreover, researching bigger samples can yield valuable outcomes that can be used to notice and analyse this effect in the presence of influential organizational factors. However, it is possible that the research sample may not adequately capture the impact of organizational factors, potentially limiting the study’s precision in this area. Nevertheless, the findings have broader applicability beyond higher education institutions, extending to diverse industries such as manufacturing and services. Variations in findings may emerge when the same model is applied in different contexts. Future research should prioritize examining sectors with a homogeneous organizational culture and higher energy use intensity, as this focus would enhance the clarity and interpretability of the results.

### Managerial and policy implications

The findings in this study can help university managers identify system weaknesses (such as organizational factors) and create and implement policies and strategies to stimulate energy-saving and other pro-environmental behaviors among academics in their educational institutions. This will contribute to a more sustainable world and a better university image in the international academic community. The use of modern technology might enable the reduction of energy usage. Prior research has undertaken the construction of a smart home energy management system^77^. Universities can also develop energy management system software. Thus, from a managerial point of view, there is a need to attract financial resources and then invest in technology, new equipment and software that makes it easier for both academics and managers to adopt greener behaviors. By understanding their impact, people are more prone to change their habits.

The policy implications of our findings refer to the need for broader policies and strategies that can be developed and implemented by governments to help universities reach their sustainable development goals in terms of being more efficient and eco-friendly. General guidelines and directions established through public educational policies can be incorporated by academic managers in both public and private universities. Policies addressing the partnership between universities and public authorities for making green investments in the campuses and their office buildings are also important in offering adequate support to universities.

### Social implications

From a social perspective, the research raises awareness of the importance of adopting energy-saving behaviors in all types of organizations, including universities. Academics educate students, providing them with knowledge in various fields, but they are also models for other aspects. One of these can be environmental interest and concern exhibited by academics and by the entire university as a structure in which students learn.

Another implication is that by changing the university buildings and campuses to make them more eco-friendly, the entire community understands the importance of such behaviours for a more sustainable world and a ripple effect will be possible.

### Limitations and future research

Further research can explore the computations and software related to this topic. Additionally, the significance of implementing neuromorphic hardware is growing in research^78^. Neuromorphic processing technology enables the measurement of emotional responses^79^. Hence, future studies might explore the psychological aspects that underlie the energy behavior of academicians with this trending technology. Video Sentiment Analysis is a contemporary technique employed to comprehend human behavior. The video’s emotional content is strongly linked to human health and well-being^80^. This technology can allow for observing academic behavior according to ethical guidelines, leading to significant scientific findings on the underlying variables influencing such behavior. For future research directions, the inclusion of cultural dimensions of Hofstede^81^ and an increase in the number of respondents for each country might contribute to a more comprehensive knowledge of the behavior of academics. The limitations of our study are related to the sample, including the academics in four countries. The results do not consider the differences in their cultures, which might partially explain why some hypotheses were not validated.

Despite the limitations inherent to any research, we appreciate that our study can be helpful for other researchers studying the behavior of academics in other countries and adding new variables to the model, such as the cultural dimensions^81^. The implications deriving from our findings refer to changes that can occur at the managerial level, but also in the broader communities of those universities. The interest in more sustainable practices is high^82^and a study focused on academics is of interest if we consider the literature gap. Greener innovations in the higher education system can start by following the practices in the business sector^82^ from which academic managers can learn to be more efficient. The originality of our study consists in the added value to the body of research on this topic and in using an extended TPB model that integrates the organizational factors in the study. Another element of novelty is brought by studying energy-saving behaviors of academics in Romania, Bulgaria, Slovakia, and Turkey, which can be a starting point for future studies by other researchers.

## Data Availability

Data availability: Data supporting the results of the study can be accessed upon reasonable request from the corresponding author.

## Abbreviations

ATT: Attitude Toward Behaviour
AVE: Average Variance Extracted
CR: Composite Reliability
ESB: Energy Saving Behaviour
ESI: Energy Saving Intention
EC: Environmental Concern
EK: Environmental Knowledge
OC: Organisational Climate
OI: Organisational Identification
OS: Organisational Support
PBC: Perceived Behavioural Control
SN: Subjective Norms
TPB: Theory of Planned Behaviour
VIF: Variance Inflation Factor
